# Patterns of zolpidem use among Iraq and Afghanistan veterans: A retrospective cohort analysis

**DOI:** 10.1371/journal.pone.0190022

**Published:** 2018-01-23

**Authors:** Ramona Shayegani, Kangwon Song, Megan E. Amuan, Carlos A. Jaramillo, Blessen C. Eapen, Mary Jo Pugh

**Affiliations:** 1 VA Southern Nevada Healthcare System, North Las Vegas, Nevada, United States of America; 2 South Texas Veterans Health Care System, San Antonio, Texas, United States of America; 3 University of Texas Health Science Center San Antonio, San Antonio, Texas, United States of America; 4 Center for Health Quality, Outcomes and Economic Research, Edith Nourse Rogers Memorial Veterans Hospital, Bedford, Massachusetts, United States of America; 5 VA Salt Lake City Health Care System, Salt Lake City, Utah, United States of America; 6 University of Utah Health, Division of Epidemiology, Salt Lake City, Utah, United States of America; University of Rome Tor Vergata, ITALY

## Abstract

**Background:**

Although concern exists regarding the adverse effects and rate of zolpidem use, especially long-term use, limited information is available concerning patterns of zolpidem use.

**Objective:**

To examine the prevalence and correlates of zolpidem exposure in Iraq and Afghanistan Veterans (IAVs).

**Methods:**

A retrospective cohort study of zolpidem prescriptions was performed with National Veterans Health Administration (VHA) data. We gathered national VA inpatient, outpatient, and pharmacy data files for IAV’s who received VA care between fiscal years (FY) 2013 and 2014. The VA pharmacy database was used to identify the prevalence of long term (>30 days), high-dose zolpidem exposure (>10mg immediate-release; >12.5mg extended-release) and other medications received in FY14. Baseline characteristics (demographics, diagnoses) were identified in FY13. Bivariate and multivariable analyses were used to examine the demographic, clinical, and medication correlates of zolpidem use.

**Results:**

Of 493,683 IAVs who received VHA care in FY 2013 and 2014, 7.6% (n = 37,422) were prescribed zolpidem in FY 2014. Women had lower odds of high-dose zolpidem exposure than men. The majority (77.3%) of IAVs who received zolpidem prescriptions had long-term use with an average days’ supply of 189.3 days and a minority (0.9%) had high-dose exposure. In multivariable analyses, factors associated with long-term zolpidem exposure included age greater than 29 years old, PTSD, insomnia, Selim Index, physical 2–3 conditions, opioids, antidepressants, benzodiazepines, atypical antipsychotics, and stimulants. High dose exposure was associated with PTSD, depression, substance use disorder, insomnia, benzodiazepines, atypical antipsychotics, and stimulant prescriptions.

**Conclusion:**

The current practices of insomnia pharmacotherapy in IAVs fall short of the clinical guidelines and may reflect high-risk zolpidem prescribing practices that put Iraq and Afghanistan Veterans at risk for adverse effects of zolpidem and poor health outcomes.

## Introduction

Once the mainstay of insomnia treatment, benzodiazepine prescription rates have fallen as a result of clinical practice guidelines discouraging their use [1–4]. Subsequently, a steady increase in the use of non-benzodiazepine hypnotics, specifically zolpidem, has been observed within the U.S. Department of Veterans Affairs (VA) healthcare system and non-VA settings [2,5]. Although zolpidem is marketed as a safe alternative for treatment of insomnia, emerging data suggests that its use is associated with safety concerns resembling those seen with benzodiazepines [6].

In addition to causing cognitive impairment and dizziness along with adverse events such as complex sleep related behaviors, falls, head injuries, fractures and traffic accidents [7–11], data now shows that zolpidem is the leading psychiatric medication linked to emergency department (ED) visits with 25% requiring hospital admissions [12], in part due to co-ingestion of another CNS depressant (e.g., benzodiazepine, opioid, alcohol) [13,14]. Data from the national Drug Abuse Warning Network (DAWN) showed that the estimated number of ED visits involving zolpidem-related suicide attempts tripled from 2004 to 2011, reaching over 14,000 visits in the latter year [15]. The rates of abuse and dependency for zolpidem are comparable to benzodiazepines and are especially concerning in patients with mental health conditions and substance use disorders (SUD)[16]. On this basis, zolpidem is classified as a Schedule IV controlled substance in the U.S. along with benzodiazepines [17].

As with any sedative hypnotic agent, the risk for adverse health outcomes is especially concerning with higher doses and long-term use. In January 2003, the U.S. Food and Drug Administration (FDA) issued a drug safety communication to lower the recommended dose for zolpidem products partly because of the lingering next-day psychomotor and cognitive effects for women and older adults who physiologically eliminate zolpidem more slowly due to the increased half-life [18,19]. Although some studies have demonstrated repeated nightly use to be safe and effective for up to one year [20–22], zolpidem is recommended for short-term use to temporarily relieve symptoms of insomnia [23]. However, the specific period of short-term use has not been delineated. Nevertheless, there is growing anecdotal evidence that zolpidem is routinely used contrary to FDA and manufacturer recommendations despite the greater awareness of its potential risks of harm [24–26].

Iraq and Afghanistan war veterans (IAVs) may be particularly vulnerable to zolpidem exposure given their behavioral and medical risk factors for adverse health outcomes, including: suicide, accidental overdose from prescription medications, and motor vehicle accidents [27–30]. However, no prior work to our knowledge has examined the extent to which zolpidem is used contrary to FDA and manufacturer recommendations in the IAV population. Thus, we aimed to describe the prevalence, duration, and mean daily dose of zolpidem prescriptions among a national cohort of IAVs, in addition to identifying key patient sociodemographic and clinical factors associated with these prescription patterns.

## Methods

### Design

This retrospective cohort study was approved by the Institutional Review Boards at the University of Texas Health Science Center at San Antonio and the Edith Nourse Rogers Memorial Veterans Hospital; a waiver of informed consent was granted prior to initiation.

### Population

We first identified IAVs using the national Operations Enduring and Iraqi Freedom and New Dawn (OEF/OIF/OND) roster file, which is provided by the VA Office of Public Health. This roster identifies individuals who were deployed in support of combat operations in Iraq and Afghanistan or provided direct support from outside the designated combat zones and who were discharged from military service (active duty) or who returned from deployments (Reserve and National Guard) prior to the end of 2011. Those IAVs that accessed VA inpatient or outpatient care at least once annually in fiscal year (FY) 2013 and 2014 (October 1, 2012 to September 30, 2014) were selected for inclusion.

### Data sources

We obtained VA inpatient and outpatient administrative data using the national VA data repository in Austin, Texas, and pharmacy records from the VA Pharmacy Benefits Management Strategic Health Group. These national data sources were then linked to the OEF/OIF/OND roster using an encrypted identifier, consistent for each individual across all databases. Prescriptions for zolpidem and other medications were identified in FY 2014 and baseline demographic characteristics and comorbid conditions were identified in FY 2013.

## Measures

### Study outcome definitions

The main study outcomes were related to zolpidem prescriptions in FY 2014. We first identified all individuals who were dispensed any zolpidem prescriptions based on the generic drug name. *Duration of treatment* was calculated by adding the days’ supply of zolpidem dispensed during FY 2014. The *average daily dose* was computed using the following formula:

$$\left(\frac{zolpidem\;dose}{pill}\right)\;\left(\frac{quantity\;of\;pills\;dispensed}{total\;prescription\;days^{\prime }supply}\right)$$

Because there is no consensus in the literature on what constitutes long-term zolpidem treatment, we considered zolpidem exposure as long-term if prescriptions were dispensed for more than 30 days because clinical trials found zolpidem treatment to be clinically significant for only four weeks and the FDA approval is for short-term use [23]. High-dose exposure was defined by an average daily dose exceeding 12.5mg for extended-release formulations and 10 mg for immediate-release formulations based on the latest FDA warning [18].

### Sociodemographic covariates

We obtained date of birth, sex, race/ethnicity, and educational attainment using the OEF/OIF/OND roster and supplemented with VA data when missing. Age was based on the first day of FY 2013 (October 1st, 2012) and was classified as follows: 18 to 29 years, 30 to 39 years, 40 to 49 years, and 50 years and older. Race/ethnicity was categorized as African American, Asian, Hispanic, Native American/Pacific Islander, non-Hispanic White, and unknown. Education at the time of discharge included less than high school, high school graduate, some college, college or higher degree graduate, and unknown. We obtained marital status (married vs not married) using VA inpatient and outpatient data in FY 2013.

### Comorbid condition covariates

We used *International Classification of Diseases*, *Ninth Revision*, *Clinical Modification (ICD-9-CM)* codes from national VA inpatient and outpatient data to characterize baseline psychiatric and medical comorbidities in FY 2013 as dichotomous variables (yes/no). We used a validated approach to identify chronic conditions (except for TBI- see below) that required *ICD-9-CM* diagnosis codes based on a minimum of one inpatient clinical encounter or two outpatient clinical encounters at least seven days apart [31]. Based on guidance recommending clinicians code TBI only on the first visit [32], TBI was based on a single inpatient or outpatient diagnosis. Conditions that are prevalent in IAVs and may be associated with zolpidem prescriptions were identified, including: TBI, PTSD, depression, SUD, anxiety, headache, pain other than headache, insomnia, chronic pulmonary disease, and sleep apnea. Finally, the Selim physical comorbidity index (excluding back pain and chronic pulmonary disease) was calculated to measure the burden of medical conditions [33]. Due to non-normal distribution, we classified comorbidity count as zero, one, two to three, and four or more.

Because Central Nervous System (CNS) polypharmacy is common among IAVs, with zolpidem as a common contributor [34], the following VA medication classes prescribed in FY 2014 were identified: antidepressants, benzodiazepines, stimulants, opioid analgesics, atypical antipsychotics, and sedating antihistamines. We classified each medication by days’ supply (e.g., 0, 1–30, 31–60, 61–90. 91–180, and more than 180 days); however, this did not result in any significant differences. Therefore, the CNS acting medications were summed up as any or no use.

### Statistical analysis

Bivariate analyses using the χ^2^ statistic were performed to describe characteristics of individuals with and without zolpidem prescriptions, and those with and without long-term and high-dose zolpidem exposure. Multivariable logistic regression analyses were used to identify demographic characteristics, comorbidities, and medications associated with receipt of: 1) any zolpidem prescriptions, 2) long-term, and 3) high-dose exposures. Results are reported as adjusted odds ratios (AORs) with 95% confidence intervals (CI). All statistical analyses were conducted using SAS version 9.3 software^®^ (SAS Institute, Inc., Cary, North Carolina); *P* < 0.05 was used as the level of statistical significance.

## Results

**Fig 1:**
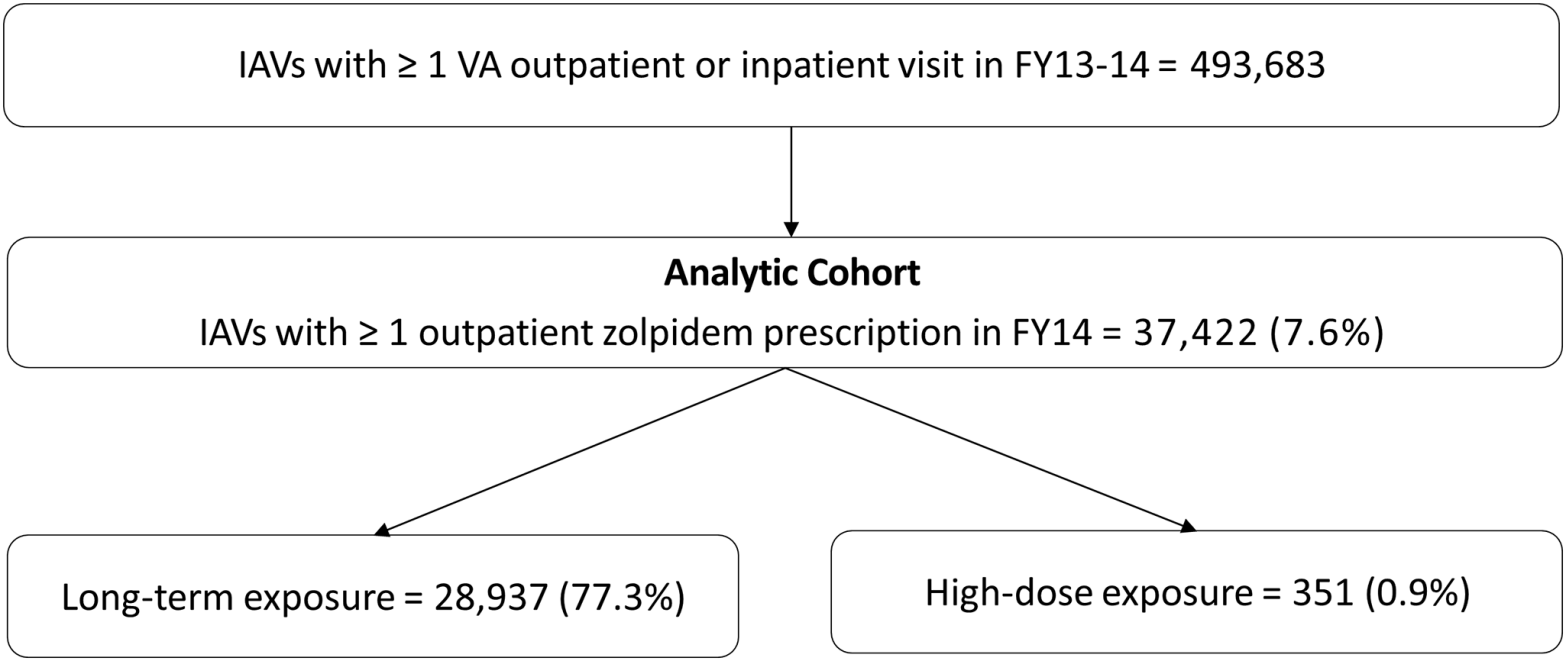
Derivation of study cohort of Iraq and Afghanistan veterans with zolpidem exposure in fiscal year 2014.

Of the 493,683 individuals who received VA care in FY 2013 and FY 2014, 37,422 (7.6%) received zolpidem. Of those who received zolpidem, 28,937 (77.3%) had long-term exposure and 351 (0.9%) received high-dose zolpidem (Fig 1).

### Any zolpidem exposure

Table 1 shows sociodemographic and clinical characteristics for those with and without zolpidem prescriptions, and adjusted odds ratios (AOR) from logistic regression predicting zolpidem exposure in FY14. For those with zolpidem prescriptions dispensed, the mean daily dose was 8.2 ±2.4mg for immediate-release (IR) zolpidem and 11.4 ±2.4mg for extended-release (ER) zolpidem.

**Table 1:** Adjusted odds ratios (AOR) of correlates of zolpidem use among Iraq and Afghanistan veterans in fiscal year 2014.

Characteristics		Zolpidem		No zolpidem		AOR	95% CI
		n = 37,422	7.6%	n = 455,027	92.4%		
Zolpidem daily dose, mg	Immediate release, mean ±SD	8.2	±2.4	−	−	−	−
	Extended release, mean ±SD	11.4	±2.4	−	−	−	−
Age, years	Mean ±SD	38.1	±9.6	37.4	±9.9	−	−
	Under 29	7,309	19.5	107,286	23.6	Reference group	
	30–39	15,612	41.7	184,917	40.6	**0.96**	**0.92–0.99**
	40–49	9,050	24.2	96,082	21.1	**1.06**	**1.01–1.11**
	50+	5,451	14.6	66,742	14.7	1.00	0.95–1.06
Sex	Men	32,180	86.0	393,109	86.4	0.97	0.93–1.01
Race/ethnicity	White	24,503	65.5	290,733	63.9	Reference group	
	Black	5,880	15.7	87,706	19.3	**0.92**	**0.88–0.95**
	Asian	932	2.5	11,835	2.6	**1.13**	**1.04–1.24**
	Hispanic	5,228	14.0	53,317	11.7	**1.12**	**1.07–1.17**
	Native American/Pacific Islander	649	1.7	6,856	1.5	1.10	0.99–1.23
	Unknown	230	0.6	4,580	1.0	**0.82**	**0.70–0.98**
Level of education	Less than high school	436	1.2	5,482	1.2	1.00	0.85–1.10
	High school graduate	29,259	77.9	354,772	78.0	Reference group	
	Some college	3,775	10.1	44,850	9.9	**1.07**	**1.02–1.12**
	College or higher degree	3,519	9.4	44,002	9.7	**1.23**	**1.17–1.29**
	Unknown	533	1.4	5,921	1.3	**1.19**	**1.06–1.34**
Marital status	Married	19,119	51.1	208,348	45.8	**0.94**	**0.92–0.97**
Comorbidities	Traumatic brain injury	10,744	28.7	74,329	16.4	0.99	0.95–1.02
	Posttraumatic stress disorder	26,331	70.4	177,876	39.1	**1.30**	**1.26–1.35**
	Depression	22,507	60.1	151,622	33.3	1.03	1.00–1.07
	Substance use disorder	9,466	25.3	80,500	17.7	**0.81**	**0.78–0.84**
	Anxiety	14,259	38.1	99,347	21.8	1.01	0.97–1.04
	Headache	12,629	33.8	87,930	19.3	**1.06**	**1.02–1.09**
	Other pain	27,745	74.1	259,959	57.1	**1.09**	**1.06–1.13**
	Chronic pulmonary disease	2,821	7.5	25,116	5.5	0.97	0.92–1.03
	Sleep apnea	4,081	10.9	29,946	6.6	1.00	0.96–1.05
	Insomnia	12,888	34.4	46,793	10.3	**1.89**	**1.83–1.95**
Selim index, physical	None	19,089	51.0	281,503	61.9	Reference group	
	1 Condition	10,296	27.5	106,192	23.3	**1.04**	**1.01–1.08**
	2–3 Conditions	6,627	17.7	57,865	12.7	1.00	0.96–1.05
	4+ Conditions	1,410	3.8	9,467	2.1	0.97	0.89–1.05
Zolpidem use	FY13 + FY14	24,869	66.5	15,277	3.4	**1.33**	**1.29–1.38**
Medications in FY14	Antidepressants	28,667	76.6	163,393	35.9	**2.90**	**2.80–3.00**
	Benzodiazepines	11,535	30.8	45,869	10.1	**1.56**	**1.51–1.62**
	Stimulants	2,642	7.1	12,931	2.8	**1.50**	**1.41–1.59**
	Opioids	12,250	32.7	75,975	16.7	**1.33**	**1.29–1.38**
	Atypical antipsychotics	6,266	16.7	29,843	6.6	**1.06**	**1.01–1.10**
	Sedating antihistamines	5,192	13.9	28,096	6.2	**1.22**	**1.17–1.28**

FY: Fiscal Year; SD: Standard Deviation; TBI: Traumatic Brain Injury; SCI Spinal Cord Injury

### Sociodemographic characteristics

Blacks and unknown races had significantly lower odds than whites for receiving zolpidem while Hispanics and Asians had higher odds. Zolpidem exposure increased with education above the high school level and in unmarried individuals.

#### Comorbid and medication characteristics

Logistic regression analysis indicated that IAVs with PTSD, headache, other pain, and insomnia had higher odds of zolpidem exposure than individuals without these comorbidities. Decreased odds of zolpidem exposure was associated with SUD. Finally, IAVs who were prescribed opioids, antidepressants, benzodiazepines, atypical antipsychotics, sedating antihistamines, or stimulants in FY14 also had higher odds of receiving any outpatient zolpidem prescription.

### Long-term zolpidem exposure

Individuals with long-term zolpidem exposure had a mean daily dose of 8.4 ±2.3mg IR zolpidem and 11.4 ±2.3mg ER zolpidem while those with short-term zolpidem exposure had a mean daily dose of 7.6 ±2.6mg IR zolpidem and 10.6 ±2.9mg ER zolpidem. Table 2 shows the socio-demographic and clinical characteristics for individuals with long-term zolpidem use and AORs from logistic regression analysis predicting long-term zolpidem exposure. IAVs 30 years and older had higher odds of long-term zolpidem exposure. On the contrary, those with Black, Asian, or Native American/Pacific Islander racial backgrounds had lower odds of long-term zolpidem exposure. Individuals with PTSD, insomnia, and two to three physical comorbidities had significantly higher odds of long-term zolpidem exposure while IAVs with SUD had lower odds. Regarding medications, individuals who also received opioids, antidepressants, benzodiazepines, atypical antipsychotics, or stimulants had significantly higher odds of long-term zolpidem exposure. Also, Veterans with zolpidem use in FY13 and FY14, and individuals with high dose zolpidem in FY14 had significantly higher odds of long-term zolpidem use in FY14.

**Table 2:** Adjusted odds ratios (AOR) of correlates of long-term zolpidem use among Iraq and Afghanistan veterans in fiscal year 2014.

Characteristics		Long-term		Short-term		AOR	95% CI
		n = 28,937	77.3%	n = 8,485	22.7%		
Zolpidem daily dose, mg	Immediate release, mean ±SD	8.4	±2.3	7.6	±2.6	−	−
	Extended release, mean ±SD	11.4	±2.3	10.6	±2.9	−	−
Age, years	Mean ±SD	38.6	±9.6	36.6	±9.1	−	−
	Under 29	5,185	17.9	2,124	25.0	Reference group	
	30–39	11,889	41.1	3,723	43.9	**1.11**	**1.03–1.19**
	40–49	7,326	25.3	1,724	20.3	**1.44**	**1.32–1.57**
	50+	4,537	15.7	914	10.8	**1.57**	**1.40–1.76**
Sex	Men	24,918	86.1	7,262	85.6	0.99	0.91–1.07
Race/ethnicity	White	19,137	66.1	5,366	62.2	Reference group	
	Black	4,317	14.9	1,563	18.4	**0.80**	**0.74–0.86**
	Asian	675	2.3	257	3.0	**0.77**	**0.66–0.90**
	Hispanic	4,154	14.4	1,074	12.7	1.04	0.96–1.12
	Native American/Pacific Islander	480	1.7	169	2.0	**0.79**	**0.65–0.96**
	Unknown	174	0.6	56	0.7	0.82	0.60–1.14
Level of education	Less than high school	334	1.2	102	1.2	1.02	0.80–1.29
	High school graduate	22,366	77.3	67,930	80.1	Reference group	
	Some college	3,006	10.4	769	9.1	1.00	0.91–1.10
	College or higher degree	2,818	9.7	701	8.3	1.03	0.93–1.13
	Unknown	413	1.4	120	1.4	0.98	0.79–1.22
Marital status	Married	15,251	52.7	3,868	45.6	**0.91**	**0.86–0.96**
Comorbidities	Traumatic brain injury	8,495	29.4	2,249	26.5	1.00	0.93–1.06
	Posttraumatic stress disorder	20,849	72.1	5,482	64.6	**1.07**	**1.01–1.14**
	Depression	17,952	62.0	4,555	53.7	1.04	0.99–1.11
	Substance use disorder	7,301	25.2	2,165	25.5	**0.86**	**0.81–0.91**
	Anxiety	11,368	39.3	2,891	34.1	1.04	0.99–1.10
	Headache	10,018	34.6	2,611	30.8	1.00	0.94–1.06
	Other pain	21,923	75.8	5,822	68.6	1.04	0.98–1.11
	Chronic pulmonary disease	2,234	7.7	587	6.9	0.92	0.83–1.02
	Sleep apnea	3,339	11.5	742	8.8	1.03	0.94–1.13
	Insomnia	10,336	35.7	2,552	30.1	**1.12**	**1.06–1.19**
Selim index, physical	None	14,255	49.3	4,834	57.0	Reference group	
	1 Condition	8,022	27.7	2,274	26.8	0.96	0.90–1.02
	2–3 Conditions	5,482	18.9	1,145	13.5	**1.10**	**1.01–1.19**
	4+ Conditions	1,178	4.1	232	2.7	0.95	0.81–1.12
Zolpidem use	FY13 + FY14	7,587	26.2	4,966	58.5	**3.69**	**3.50–3.89**
	High dose in FY14	331	1.1	20	0.2	**3.22**	**2.03–5.12**
Medications in FY14	Antidepressants	31,311	83.7	34,778	92.9	**1.60**	**1.50–1.71**
	Benzodiazepines	18,162	48.5	30,795	82.3	**1.47**	**1.38–1.57**
	Stimulants	10,690	28.6	28,374	75.8	**1.49**	**1.33–1.67**
	Opioids	18,560	49.6	31,112	83.1	**1.34**	**1.27–1.43**
	Atypical antipsychotics	13,568	36.3	30,120	80.5	**1.12**	**1.04–1.21**
	Sedating antihistamines	12,556	33.6	30,058	80.3	1.04	0.96–1.12

FY: Fiscal Year; SD: Standard Deviation; TBI: Traumatic Brain Injury; SCI Spinal Cord Injury

### High-dose zolpidem exposure

Individuals with high-dose zolpidem exposure had a mean daily dose of 15.7 ±3.4mg IR zolpidem and 17.4 ±6.7mg ER zolpidem while those with low-dose zolpidem exposure had a mean daily dose of 8.2 ±2.3mg IR zolpidem and 11.3 ±2.2mg ER zolpidem. Table 3 shows descriptive statistics and AOR for logistic regression analyses predicting high-dose zolpidem exposure. Women had lower odds of high-dose zolpidem prescriptions (AOR = 0.57; 95% CI = 0.37, 0.86) compared to men. Individuals with PTSD, depression, SUD, and insomnia had significantly higher odds of receiving high-dose zolpidem. Furthermore, those Veterans who were prescribed benzodiazepines, atypical antipsychotics, and stimulants also had significantly higher odds of high-dose zolpidem exposure than individuals without these medications. Individuals with zolpidem use in FY13 had increased odds of high-dose zolpidem exposure in FY14.

**Table 3:** Adjusted odds ratios (AOR) of correlates of high-dose zolpidem use among Iraq and Afghanistan veterans in fiscal year 2014.

Characteristics		High-dose		Low-dose		AOR	95% CI
		n = 351	0.9%	n = 37,071	99.1%		
Zolpidem daily dose, mg	Immediate release, mean ±SD	15.7	±3.4	8.2	±2.3	−	−
	Extended release, mean ±SD	17.4	±6.7	11.3	±2.2	−	−
Age, years	Mean ±SD	38.6	±8.9	38.1	±9.6	−	−
	Under 29	48	13.7	7,261	19.6	Reference group	
	30–39	162	46.2	15,450	41.7	1.31	0.94–1.83
	40–49	95	27.1	8,955	24.2	1.46	0.98–2.16
	50+	46	13.1	5,405	14.6	1.17	0.72–1.89
Sex	Men	325	92.6	31,855	85.9	**0.57**	**0.37–0.86**
Race/ethnicity	White	250	71.2	24,253	65.4	Reference group	
	Black	37	10.5	5,843	15.8	0.76	0.53–1.09
	Asian		<1.0	929	2.5	0.38	0.12–1.20
	Hispanic	55	15.7	5,173	14.0	1.05	0.77–1.41
	Native American/Pacific Islander		<1.0	645	1.7	0.58	0.21–1.57
	Unknown		<1.0	228	0.6	1.00	0.24–4.06
Level of education	Less than high school		<2.0	429	1.2	1.58	0.74–3.40
	High school graduate	284	80.9	28,875	77.9	Reference group	
	Some college	33	9.4	3,742	10.1	0.94	0.65–1.36
	College or higher degree	24	6.8	3,495	9.4	0.80	0.52–1.24
	Unknown		<1.0	530	1.4	0.59	0.19–1.86
Marital status	Married	179	51.0	18,940	51.1	1.13	0.90–1.43
Comorbidities	Traumatic brain injury	136	38.8	10,608	28.6	1.04	0.82–1.32
	Posttraumatic stress disorder	315	89.7	26,016	70.2	**2.49**	**1.73–3.58**
	Depression	270	76.9	22,237	60.0	**1.57**	**1.20–2.05**
	Substance use disorder	134	38.2	9,332	25.2	**1.31**	**1.04–1.65**
	Anxiety	149	42.5	14,110	38.1	0.89	0.71–1.12
	Headache	142	40.5	12,487	33.7	1.02	0.80–1.29
	Other pain	282	80.3	27,463	74.1	0.92	0.69–1.22
	Chronic pulmonary disease	24	6.8	2,797	7.5	0.77	0.51–1.18
	Sleep apnea	57	16.2	4,024	10.9	1.28	0.95–1.73
	Insomnia	148	42.2	12,740	34.4	**1.29**	**1.03–1.60**
Selim index, physical	None	149	42.5	18,940	51.1	Reference group	
	1 Condition	107	30.5	10,189	27.5	1.12	0.86–1.45
	2–3 Conditions	80	22.8	6,547	17.7	1.20	0.88–1.62
	4+ Conditions	15	4.3	1,395	3.8	1.03	0.58–1.84
Zolpidem use	FY13 + FY14	45	12.8	12,508	33.7	**2.36**	**1.71–3.25**
	High dose in FY14	331	94.3	28,606	77.2	**3.14**	**1.98–4.98**
Medications in FY14	Antidepressants	37,363	99.8	287	76.8	0.93	0.69–1.25
	Benzodiazepines	37,236	99.5	11,721	31.3	**1.44**	**1.15–1.81**
	Stimulants	37,118	99.2	2,946	7.9	**1.74**	**1.27–2.40**
	Opioids	37,222	99.5	12,450	33.3	1.21	0.97–1.52
	Atypical antipsychotics	37,169	99.3	6,519	17.4	**1.39**	**1.08–1.78**
	Sedating antihistamines	37,125	99.2	5,489	14.7	0.93	0.69–1.25

FY: Fiscal Year; SD: Standard Deviation; TBI: Traumatic Brain Injury; SCI Spinal Cord Injury

## Discussion

We found that approximately 7.6% of IAVs were dispensed one or more zolpidem prescriptions in FY 2014 and more than three-quarters of those individuals (77.3%) had long-term exposure. A Danish study reported similar findings that approximately 94% of individuals who were prescribed Z-drugs (zaleplon, zolpidem, and zopiclone) had longer treatment exposure than the recommended four weeks [35]. Nonetheless, our finding that women were less likely to receive higher dosages is promising. This observation is consistent with a previous study that demonstrated FDA’s January 2013 Drug Safety Communication release has been effective [26], or that prescribing for women largely met the FDA criteria prior to the recommendation.

Suboptimal zolpidem prescribing practices may lead to high-risk drug interactions with serious adverse health outcomes, namely due to potentiation of CNS depressant effects [14,36–41]. We found that individuals prescribed zolpidem long-term were significantly more likely to also receive antidepressants (83.7%), benzodiazepines (48.5%), opioids (49.6%), stimulants (28.6%), or atypical antipsychotics (36.3%) prescriptions. Veterans on high-doses of zolpidem received benzodiazepine (99.5%), opioid (99.5%), and atypical antipsychotics (99.35%) prescriptions. The prescription of additional CNS acting medications is concerning given the potential for drug interactions because CNS polypharmacy is independently associated with overdose and suicide-related behaviors [34]. It is not known whether specific combinations of medications or total number of medications lead to adverse events, but this topic deserves further study.

We found that approximately 7% of the zolpidem cohort also received prescriptions for neuro-stimulants. Stimulant pharmacotherapy is commonly used for the treatment of ADHD and was recommended for the treatment of fatigue and cognitive symptoms by the 2009 VA TBI clinical practice guidelines which were in effect at the time of this study [42,43]. However, stimulants can further exacerbate sleep disturbance symptoms due to their wake-promoting effects [43]. It is not clear whether zolpidem is prescribed for insomnia secondary to ADHD and TBI or as part of a "*prescribing cascade*,” treating the undesired effects of stimulant medications [44].

Adverse reactions with zolpidem include abnormal thinking and behavioral changes which could complicate the diagnostic picture regarding depression and PTSD [45]. In this study, depression and PTSD were consistently associated with all aspects of zolpidem exposure. Surprisingly, we found that the likelihood of high-dose zolpidem exposure was significantly greater for individuals with PTSD than those with insomnia This reflects the fact that zolpidem is recommended as a second-line treatment option for management of sleep disturbances in patients with PTSD [46]. However, since psychiatric disorders such as PTSD and depression carry an inherent risk for overdose death on their own [47,48], the FDA warning for zolpidem regarding an increased risk of worsening depression or suicidality should be considered [23]. This risk can be further potentiated with the addition of high-dose or long duration zolpidem treatment to existing regimens of antidepressants and benzodiazepines [49].

Sedative-hypnotic abuse is commonly seen in individuals with substance use disorders [50,51]. Although initial zolpidem clinical trials reported a lack of abuse and dependence potential, the emerging evidence from epidemiological studies and post-marketing surveillance show that individuals with mental health conditions and substance use disorders are at higher risk for misuse of prescribed zolpidem [52–54]. In this study, it is reassuring that the zolpidem exposure was less likely for those with substance use disorder (SUD). However, among those who had zolpidem exposure, SUD was associated with high-dose zolpidem use. This may suggest suboptimal prescribing practices, tolerance (i.e., physical dependence) to the sedating effects, or drug seeking behavior to alleviate withdrawal effects or enhance the effects of other drugs in this cohort. This finding has clinical implications as individuals with SUDs may be using high-doses of zolpidem with other CNS depressing drugs.

We hope that clinicians consider a broad assessment of insomnia symptoms and optimize the management of the underlying conditions (e.g., sleep apnea, pain, ADHD, SUD) and substance use (e.g., stimulants, illicit drugs, alcohol) prior to initiating pharmacotherapy [55]. If a hypnotic such as zolpidem is initiated, it should be offered short-term for intermittent use and only as an adjunct to cognitive behavioral therapy for insomnia (CBT-I). CBT-I is now considered an important treatment approach for chronic insomnia and recommended as the initial treatment in current treatment practice guidelines [55–58]. Because of the limited number of CBT therapists, new models of delivering CBT for insomnia have been developed to meet the high demand [59–62]. It is important for providers to incorporate CBT to sustain improved sleep and to limit the use of hypnotics [63–65].

### Strengths and limitations

The present study had several strengths. To our knowledge, it is the first national-level study investigating the prevalence of zolpidem use and its prescribing patterns among IAVs. However, several limitations should be noted. Our data represents only IAVs enrolled in the VA healthcare system; thus, our results may not be generalized to all OEF/OIF/OND veterans, other veteran groups, or the U.S. general population. Because the current study used VA administrative data obtained from veterans’ medical records in a retrospective manner, our estimates of medications and diagnoses may be conservative as outside care was not included. Additionally, medication adherence and the use of "as needed" therapy cannot be confirmed. Although our models adjusted for important demographic and clinical covariates, our results may be confounded by other variables not captured in the analysis such as disease severity and the use of non-pharmacological approaches (e.g., psychotherapy). Lastly, given that patients with consistent utilization of VA services (at least one annual visit in FY 2013–2014) were included, we may have inadvertently selected for veterans with poorer health status compared to that of the OEF/OIF/OND general population. However, our cohort included about 80% of those who had VA care in FY 2014. Despite these limitations, our results elucidate that the prevalent use of zolpidem is associated with higher-risk prescribing patterns in IAV population, particularly those veterans with PTSD or on CNS activating medications. Future studies with trajectory-based models are needed to assess the potential adverse clinical outcomes associated with these prescribing patterns.

## Conclusions

As benzodiazepines have fallen out of favor due to safety concerns, an apparent trend towards zolpidem prescribing for treatment of insomnia has become increasingly widespread. The current study found that zolpidem use is common and approximately 80% of IAVs who were prescribed zolpidem had long-term exposure. Additionally, both high-dose and long-term zolpidem exposure were consistently associated with PTSD and CNS polypharmacy (e.g., benzodiazepines and opioids) which may suggest high-risk prescribing practices and subsequent increased risk of adverse health outcomes in this population. We believe our findings can inform the development of future clinical resources and treatment algorithms to guide providers in the optimal dosing and monitoring of zolpidem treatment.
